# The Laboratory and Clinical Perspectives of Magnesium Imbalance

**DOI:** 10.7759/cureus.49835

**Published:** 2023-12-02

**Authors:** Siti Nadirah Ab Rahim, Nani Nordin, Wan Farhana Azwanee Wan Omar, Sarah Zulkarnain, Santosh Kumar, Susmita Sinha, Mainul Haque

**Affiliations:** 1 Fakulti Perubatan and Kesihatan Pertahanan, Universiti Pertahanan Nasional Malaysia, Kuala Lumpur, MYS; 2 Pathology, Faculty of Medicine and Defence Health, National Defence University of Malaysia, Kuala Lumpur, MYS; 3 Periodontology and Implantology, Karnavati School of Dentistry, Karnavati University, Gandhinagar, IND; 4 Physiology, Khulna City Medical College and Hospital, Khulna, BGD; 5 Research, Karnavati School of Dentistry, Karnavati University, Gandhinagar, IND; 6 Pharmacology and Therapeutics, National Defence University of Malaysia, Kuala Lumpur, MYS

**Keywords:** laboratory methodical aspects, clinical standpoints, calcium, magnessium, magnesium disequilibrium, magnesium tolerance test, magnesium loading test, post-analytical, hypomagnesemia, hypermagnesemia

## Abstract

Magnesium (Mg^2+^) is a predominantly intracellular cation that plays significant roles in various enzymatic, membrane, and structural body functions. As a calcium (Ca^2+^) antagonist, it is imperative for numerous neuromuscular activities. The imbalance of body Mg^2+ ^ concentration leads to clinical manifestations ranging from asymptomatic to severe life-threatening complications. Therefore, the contribution of Mg^2+^ measurement regarding various laboratory and clinical aspects cannot be ignored. Mg^2+^ is often described as the forgotten analyte. However, its close relationship with body potassium (K^+^), Ca^2+,^ and phosphate homeostasis proves that Mg^2+^ imbalance could co-exist as the root cause or the consequence of other electrolyte disorders. Meanwhile, several preanalytical, analytical, and postanalytical aspects could influence Mg^2+^ measurement. This review highlights Mg^2+^ measurement's laboratory and clinical issues and some analyte disturbances associated with its imbalance. Understanding this basis could aid clinicians and laboratory professionals in Mg^2+ ^result interpretation and patient management.

## Introduction and background

Magnesium physiology

Mg^2+^ is the second most ample intracellular cation in the human being after K^+^. It is the fourth most abundant cation in the human body after sodium (Na^+^), K^+^, and Ca^2+^ [1]. A total of 99% of the body's Mg^2+^ content is mainly found in the bone (60%) and soft tissue (39%; 29% skeletal muscle, 10% other tissues like heart and liver), while a minority (1-2%) of total body Mg^2+^ is present extracellularly. Of these extracellular fluid distributions, 30% is protein-bound (primarily to albumin), and 20% forms complexes with anions like bicarbonate, citrate, sulfate, or phosphate. The remaining 50% is in an ionized form fraction, representing the biologically active form [2]. An optimum Mg^2+^ body level is crucial as an ion that plays a significant role in multiple bodily enzymatic, membrane, and structural functions [3]. Because of this, the correct interpretation of Mg^2+^ body status is as prudent as that of other essential analytes in the renal profile. However, this requires knowledge of some laboratory and clinical factors confounding Mg^2+^ measurement.

Problem statement

Because Mg^2+^ homeostasis is closely linked to other electrolytes like K^+^ and Ca^2+^, Mg^2+^ imbalance could be the consequence or the etiology of the disorders of the electrolytes mentioned above [1]. Both severe forms of hypomagnesemia and hypermagnesemia are life-threatening if not treated early. Nonetheless, the clinical importance of Mg^2+^ is frequently overlooked. 

Objectives of this study

This review highlights the laboratory and clinical aspects of Mg^2+^ measurements, including disturbances in other analytes associated with its imbalance. It aims to provide clinicians and laboratory professionals with insights into interpreting Mg^2+^ results and its utility in patient management.

## Review

Material and methods

**Figure 1:**
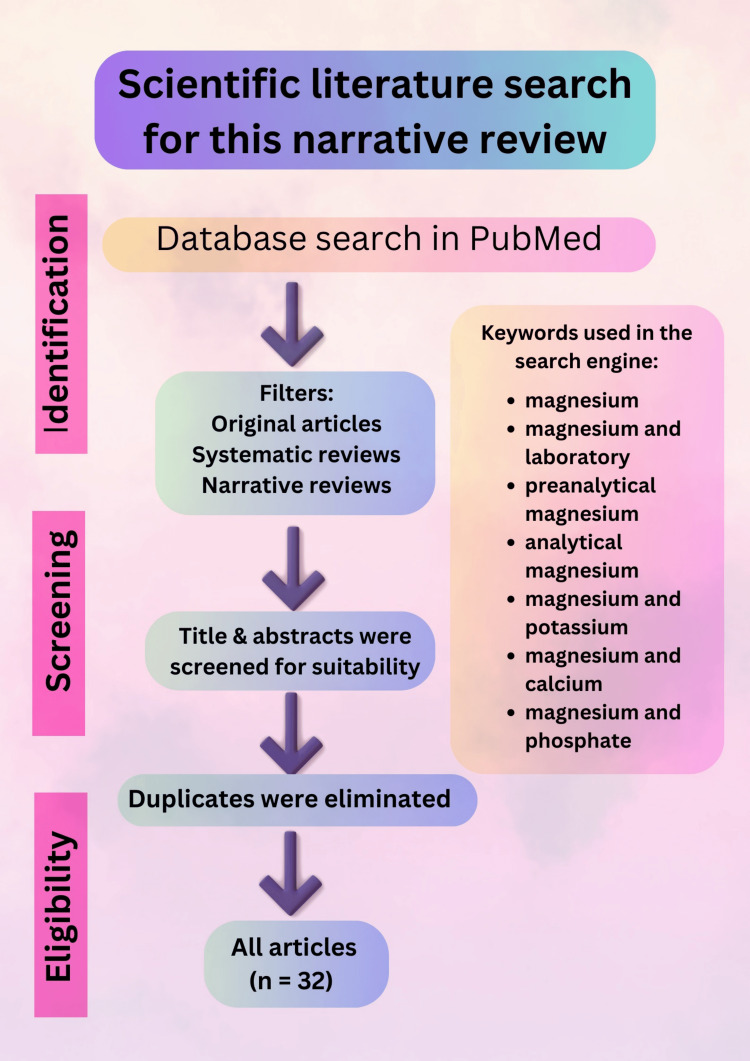
A flow chart illustrating the steps of this review paper. Note: This figure is an original work drawn by the principal author. Image credit: Siti Nadirah Ab Rahim.

This narrative review of the existing literature focuses on magnesium imbalance from laboratory and clinical perspectives. The electronic database used for article search was PubMed. The terms "magnesium," "magnesium and laboratory," "preanalytical magnesium," "analytical magnesium," "magnesium and potassium," "magnesium and calcium," and "magnesium and phosphate" were utilized in the search engine. The selected publications include original articles, systematic reviews, and narrative reviews. Duplicates were eliminated. Thirty-two papers were chosen after a rigorous assortment process based on suitability and applicability for discussing laboratory and clinical aspects of Mg^2+^. The following flow chart depicts the stage of materials and methods (Figure 1).

The roles of magnesium in the human body

**Figure 2:**
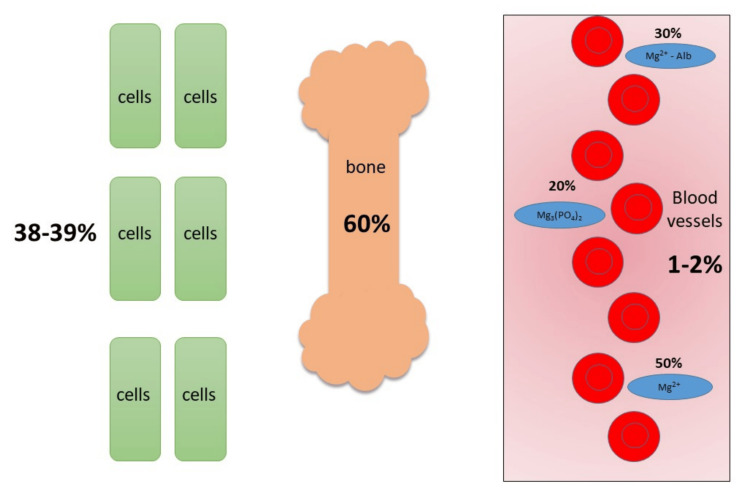
Magnesium distribution in the human body. Notes: As an intracellular cation, the majority of the body's Mg^2+^ is found in the bone (60%), followed by soft tissues (38-39%). Only 1-2% of Mg^2+^ is present in the extracellular compartment; half of this is either protein-bound or complexed with anions, and the other half exists in its free form. Mg^2+^: Magnesium; Alb: Albumin; Mg^3^(PO^4^)^2^: Magnesium phosphate. Note: This figure is an original work drawn by the principal author. Image credit: Siti Nadirah Ab Rahim.

The distribution of Mg^2+^ in the body is depicted in Figure 2.

**Figure 3:**
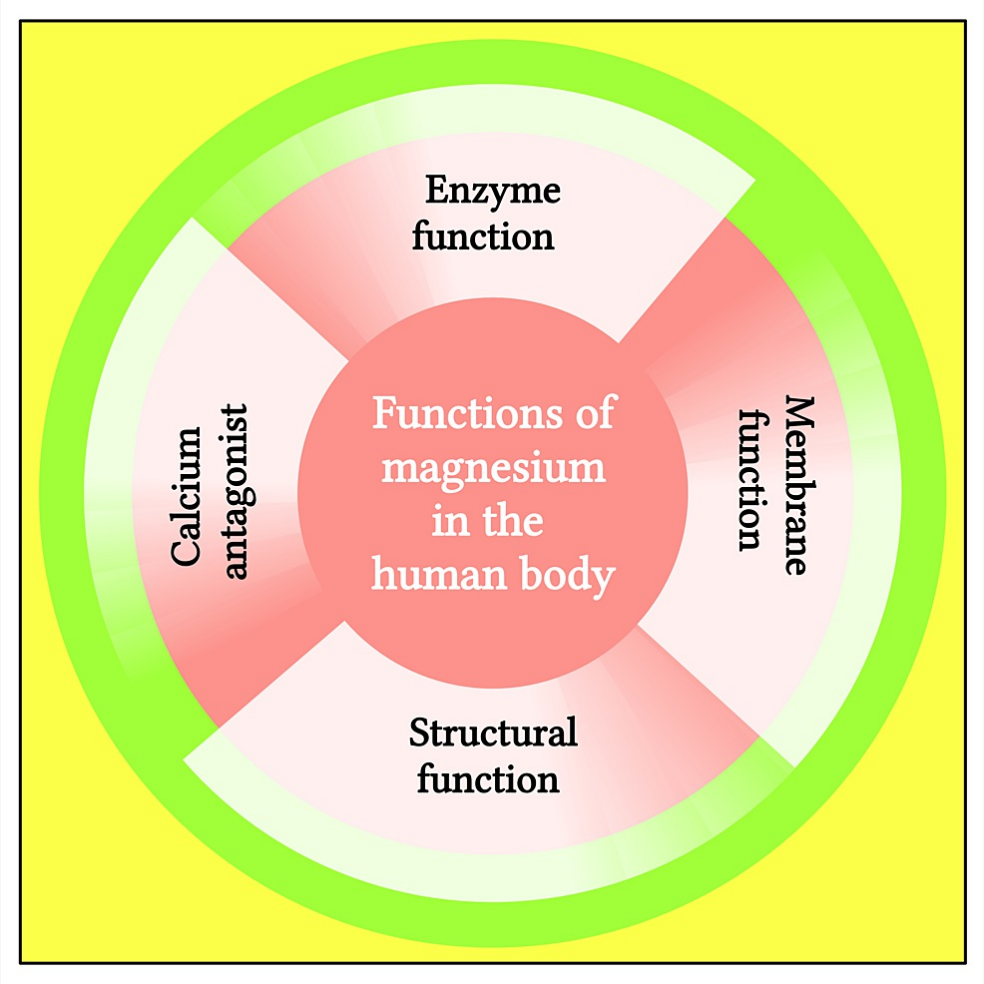
Schematic diagram showing functions of magnesium in the human body. Notes: This figure was drawn using the premium version of BioRender (https://biorender.com/, accessed on 9 November 2023) with license number UX262NVRKR. Image credit: Susmita Sinha.

Mg^2+^ has many vital physiological roles in the body (Figure 3) [3]. It acts as a Ca^2+^ antagonist pivotal in many enzymatic, membrane, and structural body functions (Table 1). 

**Table 1:** Depicting in detail the physiological roles of magnesium in the human system. Notes: Table 1 was developed based on an earlier published paper [3].

	Magnesium functions		Description
1.	Enzymatic function	Substrate for kinases, ATPases, GTPases, cyclases, activator for phosphofructokinase, creatine kinase, 5-phosphoribosyl-pyrophosphate synthetase, adenylate cyclase, and sodium-potassium ATPase (Na^+^-K^+^-ATPase)	
2.	Membrane function	Facilitates cell adhesion, aids in transmembrane electrolyte flux	
3.	Ca^2+^ antagonist	It involves muscle contractility, stimulates neurotransmitter release, mediates action potential conduction	
4.	Structural function	Forms the structure of nucleic acids, proteins, polyribosomes, enzyme structures, and mitochondria	

Mg^2+^ imbalance can manifest as hypomagnesemia or hypermagnesemia. The normal range for serum Mg^2+^ is 0.73 to 1.06 mmol/L, representing the extracellular concentration [4]. This narrative review highlights the laboratory and clinical aspects of Mg^2+^ imbalance, along with some associated analyte disturbances.

Laboratory aspects of magnesium measurement

Laboratory processes have three phases: preanalytical, analytical, and postanalytical operations [5]. The validity of analyte measurement, including Mg^2+^, depends highly on these three processes. 

Preanalytical Phase

The preanalytical variables include specimen collection site, specimen and tube selection types, specimen handling, transportation, and separation before analysis. A specimen collected at an intravenous hydration site may yield low results due to dilution. In contrast, spurious hypermagnesemia can occur if the test is performed during the concurrent infusion of Mg^2+^-containing solutions or drugs [6]. The total and free Mg^2+^ measurement can be made from serum or plasma analysis. However, in most instances, serum is preferred because anticoagulants used in plasma collection tubes might interfere with Mg^2+^ concentration. For example, a heparinized tube can significantly increase free Mg^2+^ concentration. High heparin concentration might displace the binding of Mg^2+^ with albumin; hence, the measurement of free Mg^2+^ will be substantially affected [7]. Certain silicones and thiocyanate, a product of cigarette smoke, can also interfere with free Mg^2+^ determinations. Thiocyanate interferes with the ion-selective electrode Mg^2+^ sensor [8]. In addition, supplements high in lipids, proteins, carbohydrates, various amino acids, trace elements, and different vitamin forms are also said to affect free Mg2+ measurement. Ion selective electrode (ISE) sensors should be specific enough for a particular analyte. However, in the presence of these substances, sensors' specificity towards magnesium measurement is reduced [8,9]. Anticoagulants, namely citrate, oxalate, and ethylenediaminetetraacetic acid (EDTA), are medicines used for pharmacological intervention of heavy metal-induced adverse effects and are unacceptable because of their ability to form Mg^2+^ complexes. This phenomenon will cause erroneous results in total and free Mg^2+^ measurements [6]. This is suspected if the sample exhibits concurrent hyperkalemia, hypocalcemia, hypozincemia, and low alkaline phosphatase levels [6]. The main issue during specimen handling, transportation, and separation before analysis is sample hemolysis, as this can lead to the release of intracellular Mg^2+^, hence falsely raising Mg^2+^ results, which can mask true hypomagnesemia [10, 11]. Therefore, forceful blood withdrawal, vigorous blood mixing in the tube, delayed specimen transportation, and centrifugation should be avoided to minimize erroneous results [11]. To prevent hemolysis, serum or plasma must be separated from blood cells immediately. Leakage of high RBC Mg^2+^ content in the hemolytic sample will cause a spuriously increased serum Mg^2+^ concentration, a phenomenon known as pseudo-hypermagnesemia [12].

Analytical Phase

Possible errors in this phase include interferences caused by hemolysis, elevated bilirubin, and lipemia, which can affect photometric measurements. Optimizing internal quality control and external quality assessment monitoring can efficiently detect other analytical errors. Mg^2+^ levels in the body can be determined by measuring either total or free Mg^2+^ in various samples using multiple methods.

Total magnesium measurement methods: The reference method for Mg^2+^ measurement is Atomic Absorption Spectrometry (AAS). However, photometric methods, such as the dye-binding method, are widely used in most routine laboratories. Other methods include the enzymatic method and nuclear magnetic resonance (NMR) spectroscopy [13].

(1) Dye-binding method: The basic principle is a selective binding of Mg^2+^ to metallochromic indicators, such as calmagite, chlorophosphonazo III, xylidyl blue, and arsenazo at alkaline conditions. The colored complex formed will give a spectral shift measured at a specific wavelength. The intensity of the color is proportionate to the total Mg^2+^ concentration. In this method, a Ca^2+^ chelating agent is added to reduce interference by Ca^2+^ [7,13]. For thin film reflectance photometry, adding formazan dye will form a complex with Mg^2+^ at an alkaline pH. In this method, elevated Ca^2+^ may cause a slight overestimation in Mg^2+^. This method is not interfered with by icterus, lipemia, and hemolysis [14]. 

(2) Atomic absorption spectrometry (AAS): This method provides the most accurate Mg^2+^ measurements compared to the photometric methods. The lanthanum-hydrochloric acid solution is added to reduce viscosity and interference from anions. The ground-state Mg^2+^ ions absorb light from an Mg^2+^ hollow cathode lamp. The light absorption is then measured spectrophotometrically. This is the gold standard method but is not practical for routine laboratory use [15]. 

(3) NMR spectroscopy: NMR enables the determination of intracellular Mg^2+^ by measuring the total adenosine triphosphate (ATP) that complexes with Mg^2+^. Subsequent calculations provide an estimation of free intracellular Mg^2+^. However, this method is more suitable for experimental research due to its high cost and low throughput [16].

(4) Enzymatic method: Enzymatic methods with hexokinase or other enzymes that use Mg^2+^-ATP as a substrate are also available. The rate of the enzyme-catalyzed reaction is dependent on the concentration of Mg^2+^. When glucose-6-phosphate dehydrogenase and hexokinase are used, nicotinamide adenine dinucleotide phosphate hydrogen (NADPH) formation is measured spectrophotometrically at 340 nm. Other enzymes that could be used include isocitrate dehydrogenase [14].

Free magnesium measurement using ion selective electrode (ISE): The formation of Mg^2+^ complexes in plasma is pH-dependent, and the measurement is interfered with by the presence of several analytes, including Ca^2+^ ions [7,14]. Interference by free Ca^2+^ contributes to high inter-assay variability [7]. Ionophores or electrodes demonstrate poor selectivity for Mg^2+^ over Ca^2+^. Therefore, concurrently measuring both ions is necessary to correct results that are interfered with by Ca^2+^ [7]. Generally, there is an almost equal distribution of free and bound Ca^2+^ in circulation. However, discrepancies between total and free Mg^2+^ measurements can be attributed to the redistribution of Mg2+ concentrations extracellularly and intracellularly, and to altered distributions of free and bound Ca^2+^, which occur in sick people and smokers, respectively [8,17].

Postanalytical Aspects

There is no specific postanalytical error related to Mg^2+^ measurement. Transcriptional error in reporting of unit used for Mg^2+^ (e.g., mg/dl vs mmol/L) is one of the potential errors in this phase.

**Issues in the measurement of body magnesium level** 

Intracellular vs. Extracellular Magnesium Estimation

Mg^2+^ can be measured in the serum or plasma, urine, erythrocyte, and saliva [7,15]. Nonetheless, intracellular measurement of Mg^2+^, such as in the erythrocyte, is ideally a more reliable indicator of Mg^2+^ status compared to other body fluids [7]. The ability of Mg^2+^ assays to measure Mg^2+^ in various samples allows evaluation of the total body Mg^2+^ pool. This is vital, especially when cellular Mg^2+^ concentration represents Mg^2+^ bulk in the body [7,18]. However, intracellular Mg^2+^ measurement is more experimentally relevant yet not standardized and impractical for routine use [18]. Table 2 lists the available tests for Mg^2+^ measurement [19]. Intracellular measurement is more representative of the total Mg^2+^ compared to serum. A sample of a skeletal muscle biopsy is the best due to its 30% content of all the intracellular fractions. Studies revealed that skeletal muscle biopsy better predicts cardiac muscle Mg^2+^ levels compared to lymphocyte and serum. Nevertheless, because it is invasive, expensive, and lacks the expertise to perform this procedure, it is not the method of choice for routine laboratory practice [20]. Therefore, it can be concluded that serum Mg^2+^ is still the most practical and feasible method [18].

**Table 2:** The list of available tests for magnesium measurement. Notes: Table 2 was developed based on an earlier published paper [19].

Available Tests for Magnesium Measurement
Total serum Mg^2+^
24-hour urinary Mg^2+^ and fractional excretion of Mg^2+^
Oral or IV Mg^2+ ^loading test
Intracellular Mg^2+ ^measurement in the erythrocytes, hair, muscle, and bone

Serum vs. Plasma Measurement

Serum is preferred to plasma because the anticoagulants used in plasma could be complexed with Mg^2+^. For instance, citrate binds Ca^2+^ and Mg^2+^, affecting fluorometric and colorimetric procedures [15].

Free (Ionized) vs. Total Magnesium Measurement

Twenty percent (20%) of skeletal Mg^2+^ reserve and the very minute amount of soft tissue cell reserve are exchangeable. However, it readily traverses across the membrane. Therefore, serum Mg^2+^ was previously considered a better indicator of total body Mg^2+^. Nonetheless, there is poor agreement across different assays because of poor membrane selectivity and considerable inter-assay variation [14]. Thus, total serum Mg^2+^ level is still the most convenient, widely used, and robust method in routine laboratories [15]. However, to the current date, many studies have demonstrated poor correlations between serum Mg^2+^ and intracellular levels. Therefore, serum Mg^2+^ measurement does not reliably reflect the total body Mg^2+^ content, but it is widely used for its practicality and wide availability [15]. 

Magnesium Loading Test

Mg^2+^ is primarily an intracellular ion. Hence, the next best option to approximate the total body Mg^2+^ level is the parenteral Mg^2+^ loading test, also known as a Mg^2+^ tolerance test. This test estimates the percentage of Mg^2+^ retention post intravenous Mg^2+^ load [19]. Following a baseline 24-hour urine collection, 0.1 mmol/kg body weight of Mg^2+^ is given intravenously in 5% dextrose before the 24-hour urine Mg^2+^ is sampled again [19,20]. If Mg^2+^ stores are adequate, most of the administered Mg^2+^ is excreted in 24 hours with a percentage retention of ~14%. However, in a Mg^2+^-depleted individual, retention is 85%, and those at high risk of Mg^2+^ depletion show retention of 51% [20]. It is a reliable and sensitive method for Mg^2+^ status determination but is time-consuming and inconvenient. The list of available tests for Mg^2+^ is depicted in Table 2.

Magnesium imbalance

Hypomagnesemia

Hypomagnesemia is defined as a serum Mg2+ level of less than 0.6 mmol/L but often manifests only when the value is less than 0.5 mmol/L. Mg2+ concentrations of 0.5-0.6 mmol/L indicate mild hypomagnesemia, 0.4-0.5 mmol/L indicate moderate hypomagnesemia, and ≤0.4 mmol/L indicate severe hypomagnesemia [21]. Hypomagnesemia is more common than hypermagnesemia. It occurs in 25% of hospitalized patients, especially in ICU settings, due to factors such as nutrition, diuretics, hypoalbuminemia, and the use of aminoglycosides [22]. The prevalence in the general population is up to 15% [4]. Clinically, patients may exhibit neuromuscular hyperexcitability, such as tremors, tetany, convulsions, and coma. A patient with moderate Mg2+ depletion could show electrocardiogram changes, including QRS complex widening, peaked T waves, widening of the PR interval, and diminution of T waves. In severe cases, atrioventricular arrhythmias may be observed [22]. Concurrent electrolyte disturbances, such as hypocalcemia and hypokalemia, can also be found [23]. Symptoms can appear as early as in mild hypomagnesemia, when the value reaches less than 0.5 mmol/L (Table 3).

**Table 3:** Severity of hypomagnesemia. Notes: Table 3 was developed based on an earlier published paper [21].

Severity	Levels	Signs and symptoms
Mild-Moderate	0.4-0.6 mmol/L	Neuromuscular irritability, tremor, hypocalcemia, hypokalemia, widened QRS with tall T wave in moderate depletion.
Severe	Less than 0.4 mmol/L	Tetany, nystagmus, seizure, psychosis, atrial/ventricular tachyarrhythmias

**Figure 4:**
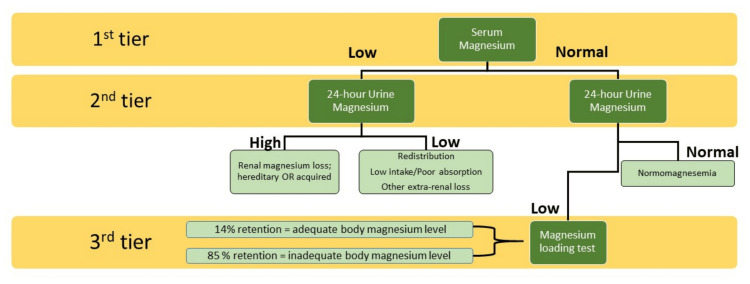
Laboratory approach to hypomagnesemia. Notes: This diagram illustrates a 3-tier approach to diagnosing hypomagnesemia and Mg^2+^ deficiency. An average serum Mg^2+^ level does not necessarily reflect the total body Mg^2+^ level. A subsequent intermediate 24-hour urine Mg^2+^ level suggests the absence of Mg^2+^ deficiency, but a low 24-hour urine Mg^2+^ level might indicate the presence of Mg^2+^ deficiency. Following the Mg^2+^ loading test, an individual with total body Mg^2+^ deficiency will retain most of the given Mg^2+^ (about 85%). To calculate the Mg^2+^ retention rate, both pre- and post-urinary Mg^2+^ level measurements are required. This figure is an original work drawn by the principal author. Image credit: Siti Nadirah Ab Rahim.

Approach to investigating hypomagnesemia: After excluding preanalytical, analytical, and postanalytical causes of abnormal Mg2+ levels, true pathological causes must be investigated (Figure 4). Serum Mg2+ is usually the initial choice of test. However, a high level would suggest renal loss if a 24-hour urine Mg2+ test is performed. This could help narrow the diagnosis to hereditary or acquired causes (Table 4).

**Table 4:** The causes of hypomagnesemia. Note: Table 4 was developed on the basis of earlier published paper [10].

Causes	Description
Decreased Intake	Low Mg^2+^ diet
	Alcoholism
	Fasting
Decreased Intestinal Absorption	Inflammatory bowel disease
	Gastrointestinal tract (GIT) malignancy
	Bariatric bypass surgery
	Short bowel syndrome
	Hypovitaminosis D
	Drugs (proton pump inhibitor)
Internal Redistribution	Refeeding syndrome
	Sepsis
	Acute pancreatitis
	Hungry bone syndrome
	Massive transfusion with citrated blood
	Chronic metabolic acidosis
	Treatment of diabetic ketoacidosis
Increased Loss	Renal
	Hereditary
	Bartter/ Gitelman syndrome (GS)
	Familial hypomagnesemia with hypercalciuria and nephrocalcinosis
	Autosomal dominant isolated hypomagnesemia
	Autosomal recessive isolated hypomagnesemia
	HNF1B mutation in early-onset diabetes mellitus
	Acquired
	Hypercalcemia in hyperparathyroidism
	Acute tubular necrosis, e.g., aminoglycosides induced
	Diuresis, e.g. Loop or thiazide diuretics, acute kidney injury resolution
	Post kidney transplant
	Hypokalemia & hyperaldosteronism *acquired or hereditary
	Uncontrolled DM
	Extra-Renal
	Prolonged large-volume diarrheal disease
	Prolonged nasogastric suction or biliary fistula combined with parenteral administration of Mg^2+^-free fluids.
	Severe Vomiting

A low level must exclude other renal causes, such as poor intake, malabsorption, excessive gastrointestinal loss, and internal redistribution [10]. There is also a condition known as normomagnesemic Mg^2+^ depletion. This occurs when there is an isolated low cellular Mg^2+^ level. Suspicion should arise in cases of refractory hypokalemia or unexplained hypocalcemia [24]. If Mg^2+^ deficiency is suspected, a 24-hour urine Mg^2+^ test can be performed; an average urine Mg^2+^ level would exclude this diagnosis. However, a low-level warrants further investigation with an Mg^2+^ loading test. If Mg^2+^ stores are adequate, up to 80% of the administered Mg^2+^ is eliminated, resulting in a retention rate of only 14% from the initial total 24-hour urine Mg^2+^ level. In contrast, a Mg^2+^-depleted individual typically retains about 85% of the administered Mg^2+^ (refer to the algorithm in Figure 2). A normomagnesemic individual will excrete more Mg^2+^ than a patient with actual hypomagnesemia after this 24-hour Mg^2+^ loading test. However, this method is not standardized and is cumbersome [18]. Alternatively, a 24-hour urinary Mg^2+^ test can be conducted to estimate body magnesium status [15].

Distinguishing between renal and extrarenal causes of hypomagnesemia can also be made using 24-hour urinary Mg^2+^. Daily urine Mg^2+^ excretion of more than 10 to 30 mg/24-hour indicates urinary Mg^2+^ wasting. Contrarily, a 24-hour urinary Mg^2+^ excretion of 10 mg or less suggests extrarenal Mg^2+^ losses [15]. Alternatively, a random urine fractional excretion of Mg^2+^ (FEMg) can also help distinguish between gastrointestinal (extrarenal) and renal loss. It is a more convenient test and less prone to inadequate sample issues in 24-hour urine collection [7,10]. Generally, a fractional excretion of Mg^2+^ of less than 2% is extrarenal origin, while above 4% is renal origin [14]. The formula for fractional excretion of Mg^2+^ (FEMg) is FEMg = [UMg X PCr] / 0.7 X PMg X UCr] X 100%. U refers to urine, and P refers to plasma or serum Mg^2+^ (Mg) and creatinine concentration. PMg is multiplied by 0.7, considering approximately 70% of extracellular Mg^2+^ is free and freely filtered by the glomerulus [1]​. 

Hypermagnesemia

Although much rarer than hypomagnesemia, hypermagnesemia is a severe electrolyte disturbance that can be fatal if not recognized and treated early. It is characterized by an Mg^2+^ level of more than 1.1 mmol/L, with symptoms manifesting at different levels of severity (Table 5).

**Table 5:** The severity of hypermagnesemia. Note: Table 5 was developed on the basis of earlier published paper [25,26].

Severity	Levels	Signs and symptoms
Mild	> 1.1 mmol/L to 3.0 mmol/L	Asymptomatic or vague symptoms of weakness, nausea, dizziness, and confusion. Usually present at concentration >2 mmol/L
Moderate	3.0 to 5.0 mmol/L	Diminished reflexes, deteriorating confusion, urinary incontinence, headache, and constipation. Hypotension, bradycardia, and blurry Vision.
Severe	5.0 to 6.0 mmol/L	Flaccid paralysis, breathing difficulty, pronounced hypotension, bradycardia, prolonged P-R interval, and atrioventricular block.
Severe to fatal	>6.0 mmol/L	Coma and cardiopulmonary arrest.

At values above 2.0 mmol/L, symptoms are typically observed and are therefore considered critical [25]. Mild hypermagnesemia is often well tolerated and may be asymptomatic. Despite its rarity, untreated or severe hypermagnesemia can be fatal [26].

*Approach to investigating hypermagnesemia*: Causes of hypermagnesemia (Table 6) could be divided into three categories: increased intake, decreased excretion, and compartmental shift [27].

**Table 6:** Causes of hypermagnesemia. Note: Table 6 was developed on the basis of earlier published paper [27].

Causes	Description
Increased intake	Amplified absorption due to poor gut motility (typical in the elderly, treatment with anticholinergics or opioids, inflammatory bowel disease patients).
	Laxatives (Mg2+ citrate), antacids (Mg2+ trisilicate), bowel preparation agents (Na+ picosulfate/Mg2+ citrate), and in treatment for eclampsia (intravenous Mg2+ sulfate).
	Extreme oral intake in chronic kidney disease patients.
	Milk-alkali syndrome (large amounts of Ca2+ and Mg2+).
	Newborns to mothers receiving Mg2+ sulfate for eclampsia.
Decreased renal excretion	Acute or chronic kidney disease.
	Adrenocortical insufficiency
	Hyperparathyroidism
	Hypercalcemia and/or hypocalciuria e.g., Familial hypocalciuric hypercalcemia (FHH).
	Lithium-based psychotropic drugs.
Compartment shift or leak	Hemolysis.
	Tumor lysis syndrome.
	Rhabdomyolysis.
	Acidosis, such as in diabetic ketoacidosis.

When suspecting hypermagnesemia in a patient, the laboratory approach includes measuring the serum Mg^2+^ level, renal profile, and estimated glomerular filtration rate (eGFR) to rule out renal impairment and any associated electrolyte disturbances, such as K^+^, Ca^2+^, and phosphate. If Familial Hypocalciuric Hypercalcemia (FHH) is suspected, a 24-hour urinary Ca^2+^ test can be conducted. Blood gases, plasma glucose, and ketones should be measured in cases of suspected diabetic ketoacidosis. Other tests may include serum creatine kinase level for rhabdomyolysis, an endocrine workup for conditions like hyperparathyroidism and adrenal insufficiency, and lithium therapeutic drug monitoring (TDM) in cases of suspected lithium overdose [4,25,27].

Clinical aspects of magnesium measurement

An Often-Overlooked Clinical Importance of Magnesium

Dietary Mg^2+^ supplementation has proven safe and convenient in disease and health [27,28]. Hypomagnesemia is increasing with age [29]. Even in health, low Mg^2+^ levels associated with aging are linked to the rapid generation of oxygen-derived free radicals, the reactive oxygen species (ROS), contributing to DNA damage, lipid peroxidation, and insulin resistance [29,30]. Studies proved that in critical care settings, hypomagnesemia is a common occurrence due to GIT and renal losses [23]. The association of hypomagnesemia with concurrent hypokalemia and hypocalcemia could lead to prolonged hospitalization, lengthy use of mechanical ventilation, extended intensive care, and high mortality among critically ill patients [10,23]. Because of these electrolytes' interrelated homeostasis, the Mg^2+^ imbalance in managing electrolyte disturbances should not be overlooked. 

Association of Magnesium and Potassium

The metabolism of K^+^ and Mg^2+^ is interrelated. Central disturbance in Mg^2+^ balance, predominantly Mg^2+^ depletion, will produce a secondary K^+^ depletion. Mg^2+^ is necessary to activate the sodium-potassium pump (Na^+^-K^+^-ATPase). The low intracellular Mg^2+^ level inhibits this pump. Hence, it depletes intracellular K^+ ^[4,23]. Meanwhile, in the thick ascending loop of Henle, intracellular Mg^2+^ provides an inhibitory effect on the renal outer medullary K^+^ (ROMK) channel [23,24]. In hypomagnesemia, this inhibitory effect is lost, impeding K^+^ reabsorption and, hence, renal K^+^ wasting. This results in concurrent hypomagnesemia and hypokalemia, which poses a high risk for developing torsade de pointes and other cardiac arrhythmias. In cases of ongoing gastrointestinal loss, alcoholism, or diuretic therapy, the hypokalemia worsens, and the patients could be refractory to K^+^ replacement therapy. Therefore, simultaneous hypomagnesemia should be suspected [24].

Magnesium and Calcium Homeostasis: Parathyroid Hormone (PTH) and Magnesium

An acute decrease in Mg^2+^ stimulates PTH secretion, while a sharp increase suppresses PTH release. This relationship is complex and partly because both Ca^2+^ and Mg^2+^ compete for transport in the thick ascending limb of the loop of Henle and bind to the human Ca^2+^-sensing receptor (CaSR) at the renal tubule and parathyroid gland level [27].

Magnesium and Calcium Homeostasis: Magnesium and Vitamin D Activity

**Figure 5:**
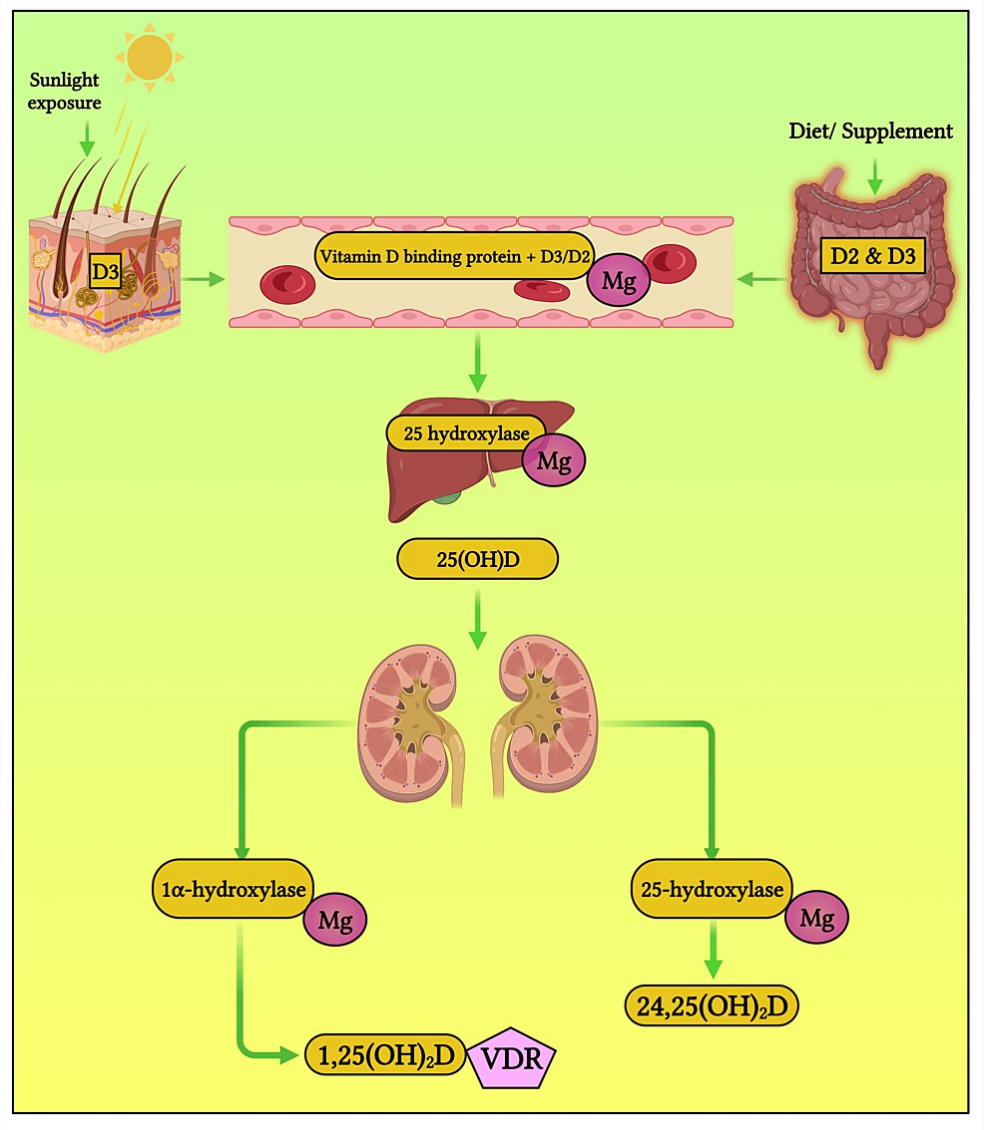
Role of magnesium (Mg) in vitamin D synthesis. Notes: D2 represents vitamin D from non-animal sources; D3 represents vitamin D from animal sources; 25(OH)D denotes calciferol (the inactive form of vitamin D); 1,25(OH)2D refers to 1,25-dihydroxy vitamin D (the biologically active state); 24,25(OH)2D is 24,25-dihydroxy vitamin D. This figure was created using the premium version of BioRender (https://biorender.com/, accessed on 9 November 2023) with license number EJ262NQ1ZQ. Image credit: Susmita Sinha.

Vitamin D metabolism relies on Mg^2+^ as the cofactor (Figure 5). Some patients with hypocalcemia and concurrent hypomagnesemia may have Mg^2+^-dependent vitamin D-resistant rickets. This is because Mg^2+^ plays a vital role in vitamin D activation. Therefore, lack of Mg^2+^ prevents vitamin D activation, hence hypocalcemia [28, 29].

Magnesium and Calcium Homeostasis: Magnesium, Calcium, Tubular Transport, and the CaSR

**Figure 6:**
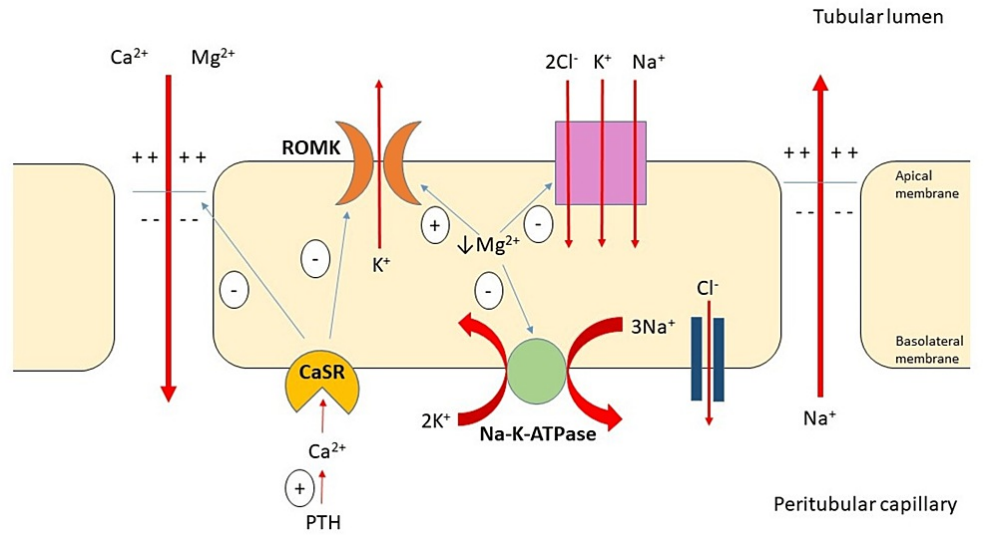
Calcium, magnesium, and K+ tubular transport at the thick ascending loop of Henle. Notes: Ca^2+^ binding to the Ca^2+^-sensing receptor (CaSR) inhibits tubular Mg^2+^ and Ca^2^+ paracellular entry and renal outer medullary K^+^ channel (ROMK) activity. This maintains the electrochemical gradient balance, preventing renal electrolyte wasting. In hypercalcemia, excessive inhibition of Mg^2+^ entry causes renal Mg^2+^ loss, leading to hypomagnesemia. Conversely, hypomagnesemia can interfere with CaSR-mediated parathyroid hormone (PTH) release, reducing PTH and Ca^2+^ levels, thereby causing hypocalcemia (not shown in the diagram). Intracellular Mg^2+^ also inhibits ROMK. A deficiency in intracellular Mg^2+^ results in the loss of ROMK inhibition, inactivity of the Na+-K+-2Cl- co-transporter, and inhibition of the Na+/K+-ATPase pump, leading to renal K+ wasting. CaSR: Ca2+-sensing receptor; ROMK: Renal outer medullary K+; PTH: Parathyroid hormone; Na+/K+-ATPase pump: Na-K-ATPase. This figure is an original work drawn by the principal author. Image credit: Siti Nadirah Ab Rahim.

Ca^2+^ binding to the CaSR causes prostaglandin and cytochrome p450 generations that inhibit ROMK. This prevents K+ secretion into the tubular lumen through ROMK, reducing K^+ ^concentration. When both urinary K^+^ and Mg^2+^ levels are low, this supports the diagnosis of refractory hypokalemia secondary to hypomagnesemia [31]. This causes inhibition of the Na^+^-K^+^-2Cl^-^-co-transporter [31,32]. In a hypercalcemic state, the CaSR binding with Ca^2+^ binding also decreases the permeability of the paracellular Ca^2^+ and Mg^2+^ pathway. The result is excessive Mg^2+^ excretion. Therefore, hypercalcemia causes hypomagnesemia, but paradoxically, hypomagnesemia will also result in hypocalcemia because of parathyroid gland-CaSR-mediated PTH inhibition [27]. This interrelationship at the renal tubular level is portrayed in Figure 6.

Association of Magnesium and Phosphate

Phosphate and Mg^2+^ are crucial for the ATP function [32]. In the kidney, hypophosphatemia frequently follows renal Mg^2+^ wasting [27]. Nevertheless, hyperparathyroidism also increases Mg^2+^ and Ca^2+^ reabsorption but reduces phosphate reabsorption at the proximal convoluted tubule [32].

Limitations of this study

This study provides a broad overview of magnesium imbalance, which the authors believe could aid in the clinical interpretation and treatment of patients with magnesium-related electrolyte disturbances. The main limitation is that PubMed was the only search engine used, and the related articles selected did not undergo detailed inclusion and exclusion criteria. Future studies could focus more on specific laboratory or clinical aspects to better suggest approaches for raising awareness of magnesium's clinical importance.

## Conclusions

Mg^2+^ is essential in the human body. Although it is often a "forgotten" analyte, its imbalance usually accompanies the imbalance of other electrolytes in a sick patient. Its narrow reference range reflects its minimal circulation concentration and high intracellular concentration. However, minute disturbance in its cellular homeostasis can result in a disturbed balance that will affect many Mg^2+^-dependent physiological activities that disrupt body functions. The laboratory should optimize preanalytical, analytical, and postanalytical processes of Mg^2+^ measurement. Clinically, Mg^2+^ imbalance should not be missed when abnormal K^+^, Ca^2+^, and phosphate levels are observed. 

## References

[1] Ray E, Mohan K, Ahmad S, Wolf MT (2023). Physiology of a forgotten electrolyte-magnesium disorders. Adv Kidney Dis Health.

[2] Ahmed F, Mohammed A (2019). Magnesium: the forgotten electrolyte-a review on hypomagnesemia. Med Sci (Basel).

[3] Mathew AA, Panonnummal R (2021). 'Magnesium'-the master cation-as a drug-possibilities and evidences. Biometals.

[4] Van Laecke S (2019). Hypomagnesemia and hypermagnesemia. Acta Clin Belg.

[5] Cadamuro J, Baird G, Baumann G (2022). Preanalytical quality improvement - an interdisciplinary journey, on behalf of the European Federation for Clinical Chemistry and Laboratory Medicine (EFLM) Working Group for Preanalytical Phase (WG-PRE). Clin Chem Lab Med.

[6] Bazzano G, Galazzi A, Giusti GD, Panigada M, Laquintana D (2021). The order of draw during blood collection: a systematic literature review. Int J Environ Res Public Health.

[7] Fiorentini D, Cappadone C, Farruggia G, Prata C (2021). Magnesium: biochemistry, nutrition, detection, and social impact of diseases linked to its deficiency. Nutrients.

[8] Zhan J, Wallace TC, Butts SJ (2020). Circulating ionized magnesium as a measure of supplement bioavailability: results from a pilot study for randomized clinical trial. Nutrients.

[9] Saad AS, Ismail NS, Gaber NS, Elzanfaly ES (2023). A chemically modified solid-state sensor for magnesium(ii) ions and esomeprazole magnesium potentiometric assay. RSC Adv.

[10] Solanki J, Runwal K, Beke N, Bahulikar A, Phalgune D (2022). Serum magnesium levels in critically ill patients on admission in ICU and its correlation with outcome. J Assoc Physicians India.

[11] Sanmartí J, Robles-Guirado JA, Jose-Cunilleras E, Bassols A (2023). Sample stability and heparin interference in ionized calcium and ionized magnesium measurements in horses using the Stat Profile Prime Plus co-oximetry electrolyte analyzer. Vet Clin Pathol.

[12] Sanandedji E, André J, Lienard A, Hentgen C, Barrans A (2022). [Hemolysis interference on clinical chemistry tests analyzed on DxC 700 AU (Beckman Coulter®) and kaliemia rendering algorithm]. Ann Biol Clin (Paris).

[13] Schutten JC, Gomes-Neto AW, Navis G (2019). Lower plasma magnesium, measured by nuclear magnetic resonance spectroscopy, is associated with increased risk of developing type 2 diabetes mellitus in women: results from a Dutch prospective cohort study. J Clin Med.

[14] Dent A, Selvaratnam R (2022). Measuring magnesium - Physiological, clinical and analytical perspectives. Clin Biochem.

[15] Reddy ST, Soman SS, Yee J (2018). Magnesium balance and measurement. Adv Chronic Kidney Dis.

[16] Somashekar BS, Ijare OB, Nagana Gowda GA, Ramesh V, Gupta S, Khetrapal CL (2006). Simple pulse-acquire NMR methods for the quantitative analysis of calcium, magnesium and sodium in human serum. Spectrochim Acta A Mol Biomol Spectrosc.

[17] Botturi A, Ciappolino V, Delvecchio G, Boscutti A, Viscardi B, Brambilla P (2020). The role and the effect of magnesium in mental disorders: a systematic review. Nutrients.

[18] Workinger JL, Doyle RP, Bortz J (2018). Challenges in the diagnosis of magnesium status. Nutrients.

[19] Razzaque MS (2018). Magnesium: are we consuming enough?. Nutrients.

[20] Swaminathan R (2003). Magnesium metabolism and its disorders. Clin Biochem Rev.

[21] Catalano A, Bellone F, Chilà D (2021). Rates of hypomagnesemia and hypermagnesemia in medical settings. Magnes Res.

[22] Negru AG, Pastorcici A, Crisan S, Cismaru G, Popescu FG, Luca CT (2022). The role of hypomagnesemia in cardiac arrhythmias: a clinical perspective. Biomedicines.

[23] Hansen BA, Bruserud Ø (2018). Hypomagnesemia in critically ill patients. J Intensive Care.

[24] Soori R, Dixit A, Tewari P (2018). Refractory hypokalemia while weaning off bypass. Ann Card Anaesth.

[25] Cascella M, Vaqar S (2023). Hypermagnesemia. https://pubmed.ncbi.nlm.nih.gov/31747218/.

[26] Yalçın Bahat P, Ayhan I, Üreyen Özdemir E, İnceboz Ü, Oral E (2022). Dietary supplements for treatment of endometriosis: a review. Acta Biomed.

[27] Aal-Hamad AH, Al-Alawi AM, Kashoub MS, Falhammar H (2023). Hypermagnesemia in clinical practice. Medicina (Kaunas).

[28] Uwitonze AM, Razzaque MS (2018). Role of magnesium in vitamin D activation and function. J Am Osteopath Assoc.

[29] Barbagallo M, Veronese N, Dominguez LJ (2021). Magnesium in aging, health and diseases. Nutrients.

[30] Wan Nik WN, Zulkeflee HA, Ab Rahim SN, Tuan Ismail TS (2023). Association of vitamin D and magnesium with insulin sensitivity and their influence on glycemic control. World J Diabetes.

[31] Castro D, Sharma S (2023). Hypokalemia. https://www.ncbi.nlm.nih.gov/books/NBK482465/.

[32] Blaine J, Chonchol M, Levi M (2015). Renal control of calcium, phosphate, and magnesium homeostasis. Clin J Am Soc Nephrol.

